# Facial Cellulitis Due to Type I Talon Cusp in a Pediatric Patient: A Case Report

**DOI:** 10.7759/cureus.34011

**Published:** 2023-01-20

**Authors:** Tulika Wakhloo

**Affiliations:** 1 Dentistry, All India Institute of Medical Sciences, Rishikesh, Rishikesh, IND

**Keywords:** pediatric preventive dentistry, facial cellulitis, maxillary lateral incisor, dental anomaly, talon cusp

## Abstract

Talon cusp is a rare odontogenic developmental anomaly with male predilection and multifactorial etiology. It projects as an accessory cusp-like structure from the cingulum area or the cement enamel junction (CEJ) affecting both primary and permanent dentition. It is imperative to clinically examine the developing dentition for occlusal harmony, especially in children with this anomaly. This report highlights facial cellulitis as a complication arising from a type I taloned maxillary lateral incisor and its endodontic management in a female pediatric patient. It emphasizes the clinical significance, early diagnosis, and prompt prophylactic management of the talon cusp so as to prevent the development of acute sequelae in children.

## Introduction

Talon cusp is a rare odontogenic developmental anomaly that projects as an accessory cusp-like structure from the cingulum area or the cement enamel junction (CEJ) of the maxillary and mandibular anterior teeth; in both primary and permanent dentition [1]. It consists of enamel and dentine with varying degrees of pulp tissue [2]. It was first described by Mitchell in 1892 [3]; thereafter, it was named Talon cusp by Mellor and Ripa due to its resemblance to an eagle’s talon [4]. Synonyms of talon cusp are supernumerary cusp, dens evaginate, tuberculated premolar, evaginated odontoma, occlusal anomalous tubercle, evaginated odontoma, and odontoma of axial core type [2,5]. 

It occurs as an isolated entity or in combination with other dental anomalies like dens invaginatus, odontome, mesiodense, supernumerary teeth, fusion, bilateral germination, cleft lip, peg-shaped maxillary incisors, and enamel clefts [6]. Hattab et al. classified talon cusp based on the varied morphological patterns as: Type 1: Talon: morphologically well-delineated additional cusp projecting from the palatal or facial surface of a primary or permanent anterior tooth extending at least half the distance from CEJ to the incisal edge, Type 2: Semi talon: an additional cusp of 1mm or more that extends less than half the distance from the CEJ to the incisal edge, and Type 3: Trace talon: enlarged or prominent cingulum and variations i.e. conical, bifid, or tubercle-like, originating from the cervical third of the root [5-7]. This classification was modified by Chin-Ying et al. based on the morphology, according to the appearance of the talon cusp as either "T," "Y," or "π" shape for major, minor, or bifid talon cusp when viewed from the incisal aspect [8].

The clinical complications caused by talon cusp are diverse. Facial or large talon cusp leads to poor esthetics. If unerupted, it resembles a compound odontoma or a supernumerary tooth causing diagnostic difficulties [7]. Soft tissue irritation during speech and mastication, occlusal interference, and displacement of teeth lead to functional problems. Pathological complications include attrition, periodontitis, dental caries, accidental cusp fracture, temporomandibular joint pain, pulpal necrosis, and periapical pathosis of the affected tooth [7,9]. In the literature, very few cases of talon cusp causing acute outcomes in children have been reported. This report highlights a case of facial cellulitis arising from a type I taloned maxillary lateral incisor and its endodontic management in a pediatric patient.

## Case presentation

**Figure 1:**
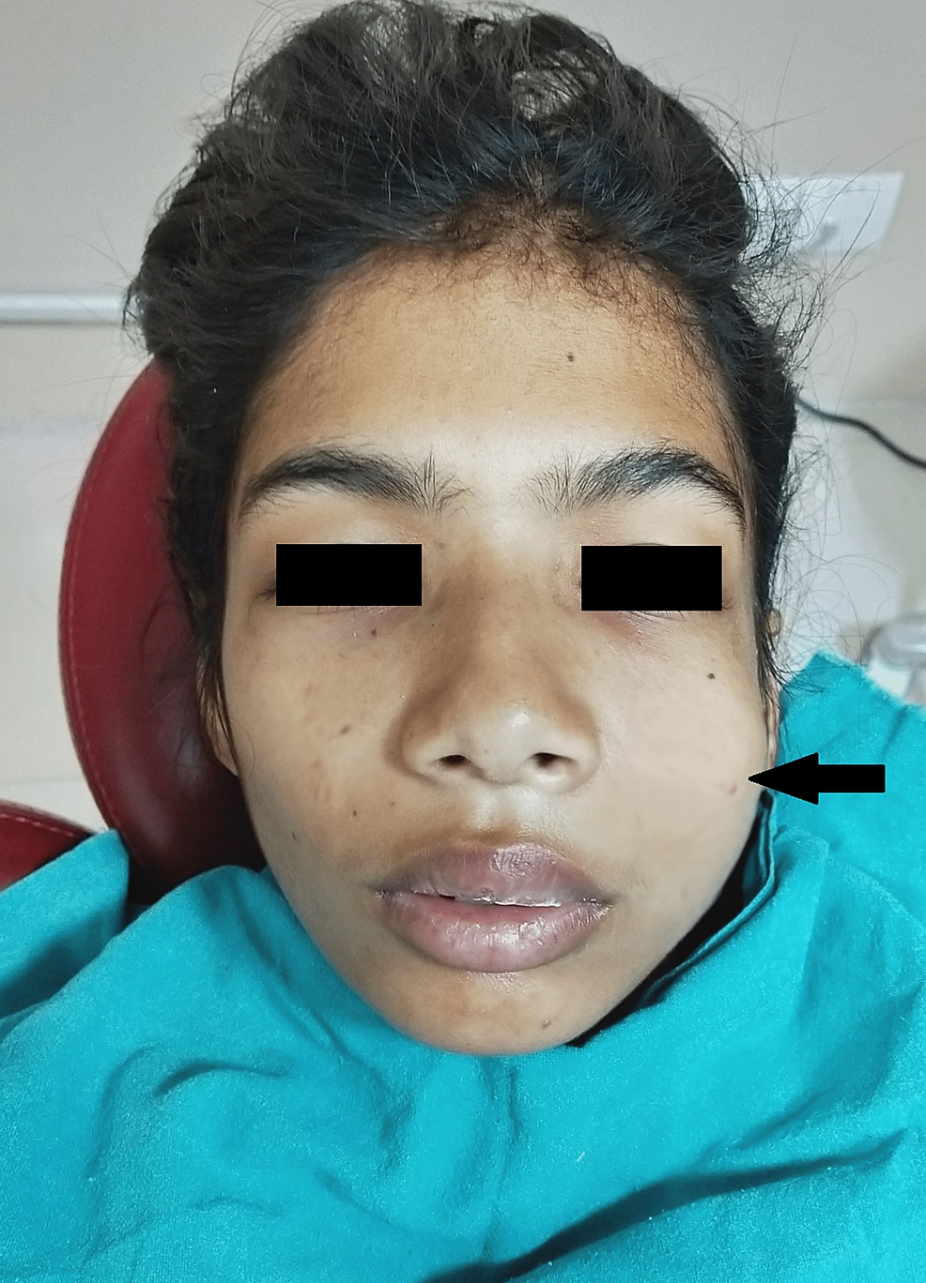
Diffuse extra oral swelling of the left side of the face

A 12-year-old female child reported to the Department of Dentistry, All India Institute of Medical Sciences, Rishikesh, India, with a chief complaint of pain in the upper left front tooth region and swelling of the face for two days. The patient also complained of a retained milk tooth in the same region. The pain was sharp, severe, spontaneous in origin, radiating to the left temporal region, and aggravated on mastication with no relieving factors. There was a history of associated low-grade fever for a day. Past medical and family histories were noncontributory. On extraoral examination, facial asymmetry with a diffuse swelling affecting the left side of the face was present (Figure 1).

**Figure 2:**
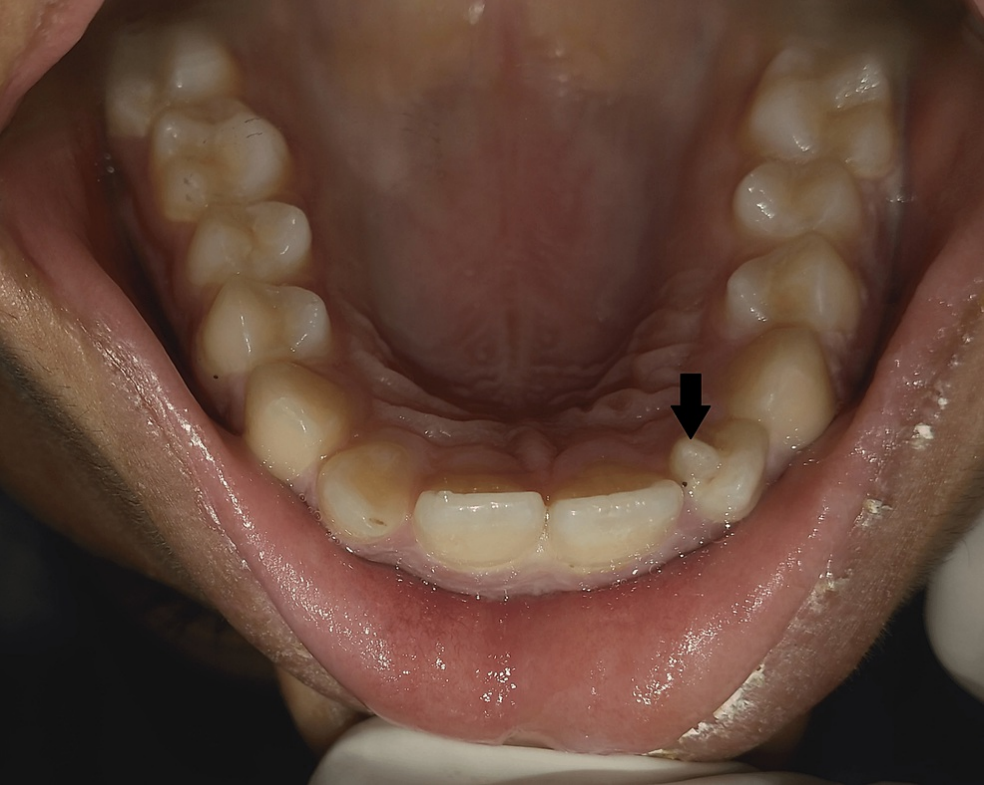
Intra-oral view of Type I talon cusp on left maxillary lateral incisor

The swelling was firm, tender on palpation with increased local temperature, and enlarged left submandibular lymph nodes. Intra-oral examination revealed permanent dentition without signs of dental caries. The retained milk tooth referred by the patient was observed to be a talon cusp on the palatal aspect of the left maxillary lateral incisor. It extended more than half the distance from the CEJ to the incisal edge and was classified as Type I talon according to Hattab et al. (Figure 2) [6].

**Figure 3:**
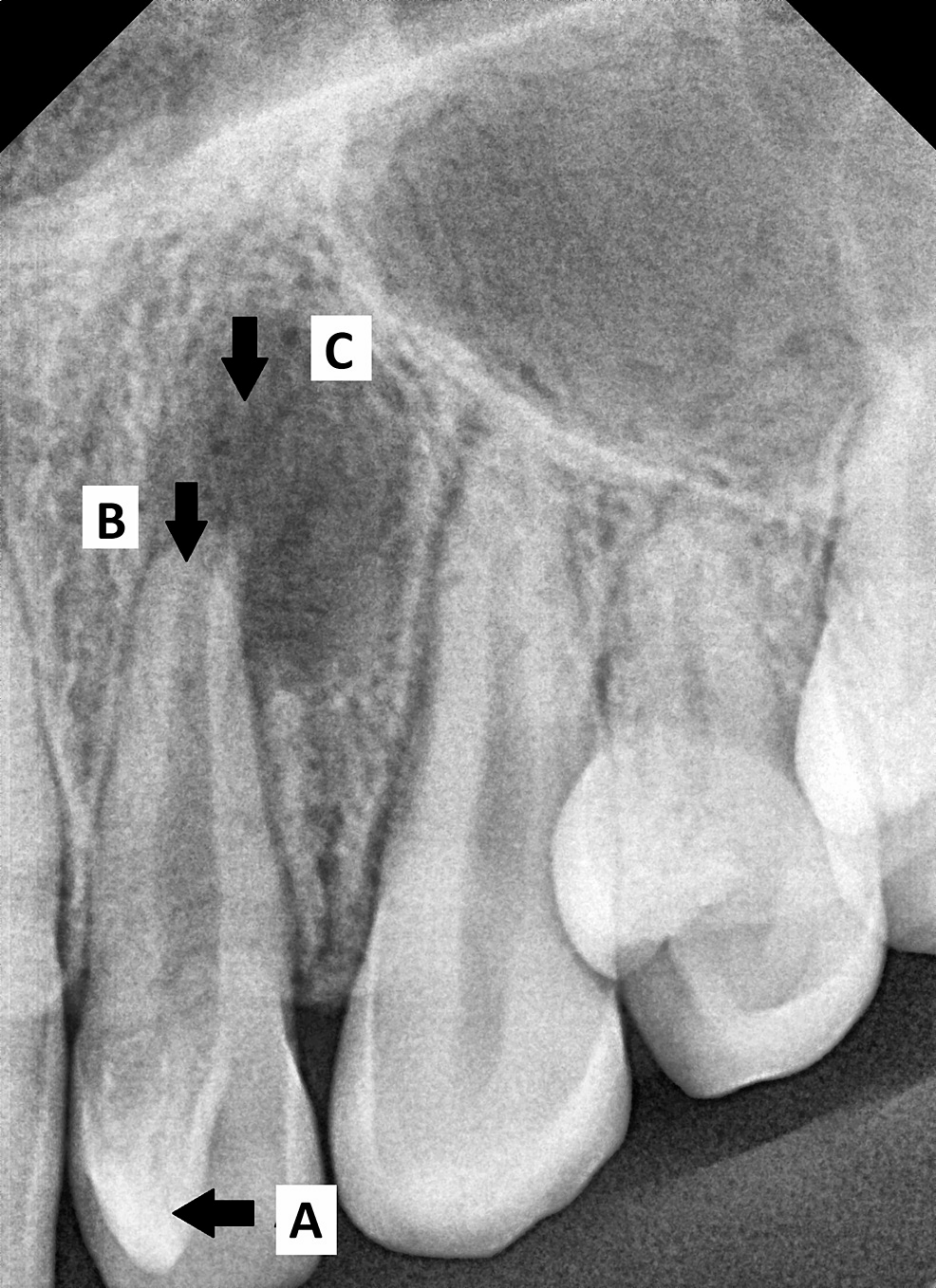
Preoperative intra-oral periapical radiograph showing (A) V-shaped radio opacity of Type I talon cusp on maxillary lateral incisor, (B) Apical root resorption, and (C) Periapical radiolucency

The left maxillary central incisor, lateral incisor, and canine were tender to apical palpation and percussion with grade I mobility. Occlusal examination revealed a class II molar relation, a rotated left maxillary lateral incisor with a T- shaped incisal edge, and a marked wear facet on the tip of the talon cusp. This was suggestive of occlusal trauma on contacting the opposite tooth. However, no occlusal interference was observed on closure. Periapical radiograph revealed a triangular or V-shaped radiopaque structure superimposed on the crown of the left maxillary lateral incisor with periapical radiolucency, widened periodontal ligament space, and open apex due to apical root resorption (Figure 3).

**Figure 4:**
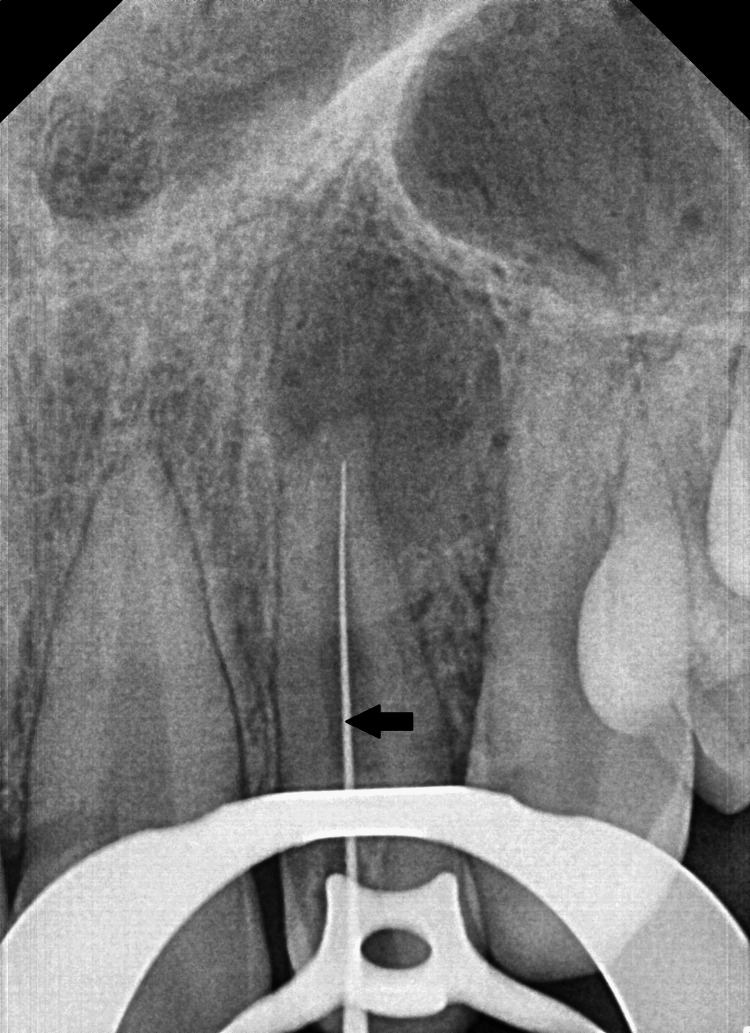
Working length determination radiograph

Based on the clinical and radiographic findings, a diagnosis of facial cellulitis due to chronic occlusal wear of Type I talon cusp with the opposing tooth was made. Non-surgical root canal treatment with apexification of the involved tooth was planned. An emergency endodontic access cavity was prepared on the palatal aspect of the left maxillary lateral incisor under rubber dam isolation and the pus was drained from the canal. The talon cusp was completely removed to relieve the occlusion followed by working length determination using the radiographic method, which was confirmed by an apex locator (Root ZX; J. Morita Corporation, Osaka, Japan) (Figure 4).

**Figure 5:**
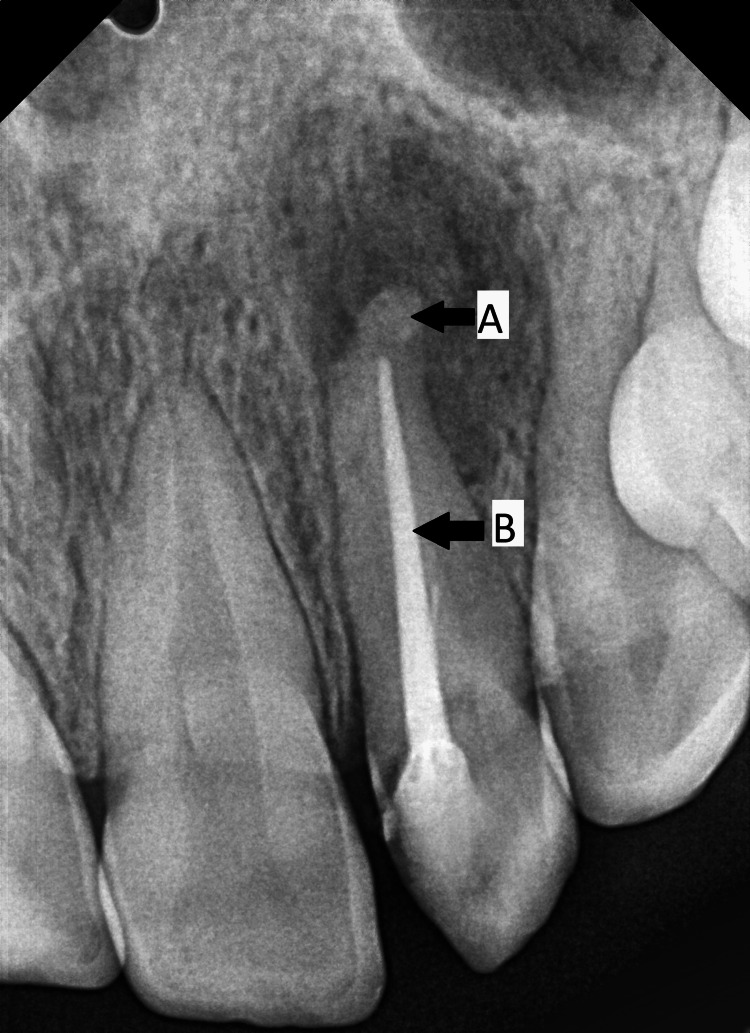
Postoperative intra-oral periapical radiograph showing (A) Apexification by formation of 3 mm apical plug by Biodentin* and (B) Obturation of the root canal * Septodont, Saint-Maur-des-Fossés, France

Biomechanical preparation was done using HyFlex® rotary files (Coltene Holding, Altstätten, Switzerland) and a lubricant (RC Prep®; Premier Dental Co., Pennsylvania, United States). The root canal was copiously irrigated with saline, 2.5% sodium hypochlorite, and 17% ethylenediaminetetraacetic acid throughout the procedure. Intracanal dressing of triple antibiotic paste was given, and the patient was advised systemic antibiotics and recalled after two weeks. On the follow-up visit, the patient was completely asymptomatic and the root canal was dry. Apexification was done by condensing Biodentine (Septodont, Saint-Maur-des-Fossés, France) using hand pluggers to form a 3 mm thick apical plug, which was confirmed radiographically. The hardness of Biodentine was evaluated after 12 minutes with a hand file followed by obturation with gutta-percha and AH 26 (Dentsply Sirona, Charlotte, North Carolina, United States) sealer using lateral condensation (Figure 5).

The access cavity was restored with universal light cure composite resin (3M™ ESPE™; 3M, Saint Paul, Minnesota, United States). The patient continued to be asymptomatic and showed improvement in periapical radiolucency after three months and was advised periodic recall.

## Discussion

Talon cusp is more common in the permanent (75%) than the primary dentition (25%) with a greater predilection in maxilla (90%) than mandible (10%) [2]. The prevalence is 0.06-10% with male predilection and a male-to-female ratio of 3:1 [5,10]. It was found to occur in 0.6% of the Mexican population, 2.5% of the Hungarian population, 5.2% of the Malaysian population, 2.4% of the Jordanian population, and 7.7% of the North Indian population [5]. The incidence in Chinese and Arab populations was reported to be higher than in Caucasian and Black populations [8]. A study found that there is no difference in the prevalence of talon cusp in the primary and permanent teeth in the Chinese population. Since talon cusps are more often than not diagnosed at the time of routine clinical examination; their prevalence in the Indian population, especially in primary dentition may be much more than reported [11]. The predominantly affected teeth are maxillary lateral incisors (55%) followed by central incisors (33%) and canines (4%) [2] with multifactorial etiology involving genetic, environmental factors, and parental consanguinity [1,9]. The susceptibility of the lateral incisors to develop this anomaly is attributed to compression of the lateral incisor tooth germ by the adjacent central incisor and canine, which develop seven months prior to the lateral incisor [12]. However, the out folding of the enamel organ or hyper-productivity of dental lamina during the morpho differentiation stage of odontogenesis is the most accepted hypothesis [1].

The best practice in pediatric dentistry asserts that the management of developing dentition and occlusion is an essential part of comprehensive oral health care. Early diagnosis and informed decisions regarding the treatment of abnormalities can lead to the attainment of stable, functional, and esthetic occlusion in children [13]. Unfavorable occlusion can cause a few cusps or a single cusp to bear the occlusal force during closure, which subsequently results in changes in the alveolar bone and periodontal connective tissue [12]. It is important to emphasize that 89.3% of dens evaginatus eventually wear out and can lead to the ingress of bacteria through immature dentinal tubules resulting in pulp necrosis [2]. In the present case, the effects of chronic occlusal wear were evident as marked wear facet on the talon cusp, apical root resorption, pulp necrosis, and resultant facial cellulitis.

Management is planned according to the chief complaint of the patient, shape and size of the talon cusp, possibility of pulp tissue involvement, and presence of other dental anomalies [2,5]. However, patients and parents may be unwilling to undergo treatment at the time of routine examination since it is commonly asymptomatic and present on the palatal aspect of teeth [2]. Prophylactic fissure sealing and preventive resin restoration can be carried out in asymptomatic cases with deep developmental grooves [2,5]. Air abrasion using a focused stream of 50-micrometer alumina particles at 60 psi for three minutes can be used for minimal preparation of the grooves [14]. In case of occlusal interference or occlusal trauma; periodic grinding at six to eight weeks intervals is recommended. This leads to the formation of reparative dentine and is followed up with the application of a desensitizing agent such as topical fluoride [5]. For guided reduction of the talon cusp, a putty index is made and adapted to teeth [14]. Furthermore, a single visit reduction of the cusp with calcium hydroxide/mineral trioxide aggregate pulpotomy or with root canal treatment can be done depending on the risk of pulp exposure. Orthodontic correction may be required in case of displacement of the involved tooth [5,8]. Considering the clinical presentation in our case, emergency access opening was done to drain the pus and relieve the patient’s acute symptoms followed by apexification and obturation of the root canal.

## Conclusions

Management of developing dentition and occlusion is an essential part of comprehensive oral healthcare in children. Facial cellulitis may be a rare complication arising from a talon cusp. Clinicians, especially pediatric dentists, can play a pivotal role in its early diagnosis along with prompt prophylactic treatment based on the initial presentation, to avoid possible complications and maintain healthy oral health status in children.

## References

[1] Gürhan C, Şener E (2017). Endodontic management of talon cusp causing apical periodontitis: a case report. J Dent Oral Biol.

[2] Saleem M, Deepa D (2016). Palatal talon's cusp of a permanent maxillary lateral incisor: a case report. Tanta Dent J.

[3] Mitchell WH (1892). Letter to the editor. Dental Cosmos.

[4] Mellor JK, Ripa LW (1970). Talon cusp: a clinically significant anomaly. Oral Surg Oral Med Oral Pathol.

[5] Gupta R, Thakur N, Thakur S, Gupta B, Gupta M (2013). Talon cusp: a case report with management guidelines for practicing dentists. Dent Hypotheses.

[6] Hattab FN, Yassin OM, al-Nimri KS (1996). Talon cusp in permanent dentition associated with other dental anomalies: review of literature and reports of seven cases. ASDC J Dent Child.

[7] Nuvvula S, Gaddam KR, Jayachandra B, Mallineni SK (2014). A rare report of mandibular facial talon cusp and its management. J Conserv Dent.

[8] Chin-Ying HS, Girija V, Fei YJ (1892). Bilateral talon cusps in primary teeth: clinical significance and treatment. ASDC J Dent Child.

[9] Ozcelik B, Atila B (2011). Bilateral palatal talon cusps on permanent maxillary lateral incisors: a case report. Eur J Dent.

[10] Prabhu RV, Rao PK, Veena K, Shetty P, Chatra L, Shenai P (2012). Prevalence of talon cusp in Indian population. J Clin Exp Dent.

[11] Kapur A, Goyal A, Bhatia S (2011). Talon cusp in a primary incisor: a rare entity. J Indian Soc Pedod Prev Dent.

[12] Segura-Egea JJ, Jiménez-Rubio A, Velasco-Ortega E, Ríos-Santos JV (2003). Talon cusp causing occlusal trauma and acute apical periodontitis: report of a case. Dent Traumatol.

[13] Management of the developing dentition and occlusion in pediatric dentistry. The Reference Manual of Pediatric Dentistry.

[14] Arora A, Sharma P, Lodha S (2016). Comprehensive and conservative management of talon cusp: a new technique. Case Rep Dent.

